# Congruence between nursing students’ and patients’ views of student–patient relationships

**DOI:** 10.1007/s10459-020-09972-z

**Published:** 2020-05-20

**Authors:** Arja Suikkala, Sanna Koskinen, Jouko Katajisto, Helena Leino-Kilpi

**Affiliations:** 1 Department of Nursing Science, University of Turku, Turku, Finland; 2 Department of Mathematics and Statistics, University of Turku, Turku, Finland; 3 Department of Nursing Science, University of Turku and Turku University Hospital, Turku, Finland; 4 Department of Nursing Science, University of Turku, FI-20014 Turku, Finland

**Keywords:** Clinical education, Cross-sectional study, Nurse–patient relations, Nursing education, Nursing students, Patient participation

## Abstract

The growing emphasis on learning with and from patients has shifted the focus from education and healthcare professionals to the student–patient relationship. The relationship between student and patient, with a supportive preceptor as a resource, can influence the progression and development of an authentic person-centred approach to care among students. The purpose of this study was to analyse the congruence between nursing students’ and patients’ views of their relationship during students’ clinical placement. The study compared data from cross-sectional matched cohort pairs of nursing students (n = 187) and patients (n = 187) in Finland. The data were collected between March 2015 and May 2016 using corresponding questionnaires and procedures in both cases. Both students’ and patients’ views were moderately or weakly congruent in terms of facilitative relationship, characterised as a mutually enriching relationship for both students and patients through dialogue. Patients, however, tended to see the relationship significantly more often as mechanistic, focusing on students learning practical skills, compared to students who saw the relationship more often as facilitative. Patients’ age and the reasons for care were the only background variables that predicted the congruence between students’ and patients’ views of their relationships. These findings suggest ways in which student–patient relationships can be made more meaningful in supporting learning in clinical education.

## Introduction

The health professional–patient relationship has been shown to contribute to positive care experiences and outcomes for patients, nurses and students (Abdolrahimi et al. 2017; Chan and Lai 2017; Feo et al. 2019). The relationship can strengthen not only patient health, but also patients’ resources for health and well-being (Strandås and Bondas 2018). Moreover, enabling patients to be active participants in their care through professional–patient relationships can improve nurses’ job satisfaction, work engagement, and disposition towards their patients (Ding et al. 2019). In clinical placements, nursing students encounter patients under the supervision of qualified staff; this results in relationships with patients that form the basis for student learning (European Commission 2005, 2013; Suikkala 2018). Both students and patients have been shown to value the beneficial consequences of the relationship in which mutual learning becomes a joint action between students and patients (Manninen 2014; Rowland et al. 2018; Suikkala and Leino-Kilpi 2005).

In a broad sense, the professional–patient relationship can be described either as a means to achieve better health outcomes, or as a shared interaction between healthcare professional and patient (Sabater-Galindo et al. 2016). The nurse–patient-relationship, specifically, has been defined as a professional, therapeutic relationship designed to enable nurses to plan, deliver and evaluate care that meets patients’ unique health needs (Feo et al. 2017). As nurse–patient-relationships form the core of nursing, the foundation of nurses’ practice is created already during their undergraduate education. Student–patient relationships across the continuum of education can help students to develop the skills and abilities they need as future professionals to provide personalised and evidence-based care to patients (Jylhä et al. 2017; WHO 2015). A growing number of initiatives focused on patient participation in health care education provide descriptions of the student–patient relationship, whereas the perspective of the student–patient relationship in the field of general nursing education in clinical practice has received very little attention (Rhodes 2012; Scammell et al. 2016; Suikkala et al. 2018).

Different relationships between health professionals and patients have been identified, such as person-oriented relationships, which are based on mutual participation between the professional and the patient, and paternalistic relationships, which are task-oriented and dominated by professionals and leave the patient outside of their own care (Kaba and Sooriakumaran 2007; Szasz and Hollender 1956). Person-oriented relationships require dialogue and can thus empower the patient through increased sense of health and well-being; these relationships focus on the patient as an equal partner and tend to transcend paternalistic practices (Castroa et al. 2016; Halldorsdottir 2008; Håkansson-Eklund et al. 2019; Uhrenfeldt et al. 2018). Due to changes in health care services and increasing amount of home- and community-based healthcare in the development of nursing education (WHO 2015), particular attention should be paid to the clinical and professional knowledge, skills and competencies needed in person-oriented care, with the aim of increasing the degree of autonomy and self-determination of patients (Henriksen et al. 2019).

The nursing student–patient relationship shares some common features with person-, professional- and task-oriented relationships—such as relationship based on dialogue between the student and the patient, or performing the planned tasks with superficial interaction (Manninen 2014; Strandås and Bondas 2018; Uhrenfeldt et al. 2018). The nursing student and patient relationship is, however, essentially different from the relationship between a qualified nurse and patient, involving a challenge in terms of balancing the patient’s caring process and the student’s learning process. Suikkala and Leino-Kilpi (2005) provided insights into the student–patient relationship from the point of view of both students and patients, revealing three types of relationship, in ascending order of reciprocity and mutuality: mechanistic, authoritative and facilitative relationships. Mechanistic relationships (MR) are externally directed by staff nurses’ routines and are focused on students’ need to acquire knowledge and technical skills. In a mechanistic relationship, students either observe nurses as role models or carry out single tasks without any dialogue with their patients. Patients are seen as passive objects who observe students’ actions and benefit from the activity. Authoritative relationships (AR) are directed by student’s perceptions of patient needs and care methods. Students practise using their expertise in planning and providing care and patient education to help patients satisfy their needs. Even if there is interaction, initiated by either party, patients prefer to receive the help and advice offered rather than expressing their opinions concerning care. Facilitative relationships (FA) are characterised by mutuality, focusing on the common good of students and patients. Through respectful dialogue, students are attentive to patients’ needs, values, and preferences, act according to them, if possible, and support patients’ use of their own resources and thus learn about how to best provide care and support. Patients, for their part, direct their own care and contribute to learning by advising students on their own health situation and by giving them feedback on their performance. (Suikkala 2007; Suikkala and Leino-Kilpi 2005). Thus, a facilitative relationship, with patient-led initiative and reciprocal knowledge-sharing, can benefit both the student and the patient, emphasising active patient participation (Suikkala et al. 2018).

As a mandatory part of nursing education, clinical training provides students with learning experiences that involve direct relationships with healthy or sick individuals and/or community, nurses, and a team of health professionals (Directive 2005/36/EC, 2013/55/EU). A competency-based curriculum can prepare nursing students for transition to work in any environment where patients access care. Thus, authentic student–patient relationships are important in meeting the unique health needs of patients and to enhance the quality of patient care (Salminen et al. 2019; Suikkala 2007). In addition to clinical placements, opportunities to develop student–patient relationships can be provided by inviting members of the public to participate in the training of nursing students (Koskinen et al. 2016).

In general, patients are happy to participate in learning relationships with students, but how they do so can vary a lot (Rowland et al. 2018; Suikkala et al. 2018). Actively participating patients can enrich and facilitate students’ authentic and meaningful opportunities for learning by sharing their knowledge and experiences—for instance, their preferred outcomes in care—and by bringing their valuable perspectives to student assessment (Castroa et al. 2016; Debyser et al. 2011; Suikkala et al. 2018). As a whole, patients’ insights on students’ professional performance provide important information about the outcomes of education (Moquin et al. 2018; Rhodes 2012). The value of patients’ meaningful influence on educational priorities is essential in preparing future professionals to work in partnership with patients, delivering high-quality person-centred care (Fong et al. 2019; Rowland et al. 2018; Scammell et al. 2016; Suikkala et al. 2018).

Student’s personal and professional attributes, patient’s attributes as a patient, and the atmosphere during collaboration shape student–patient relationships (Suikkala and Leino-Kilpi 2005; Suikkala et al. 2008, 2009). In particular, the importance of a positive and supportive atmosphere between patients, students and preceptors is highlighted by both students and patients. This often requires that students can take the time to build reciprocal collaborative relationships with patients (Castroa et al. 2016; Forber et al. 2016; Fröberg et al. 2018). Even if relationships with patients are considered important by nursing students, caring for patients engenders various emotions, such as anxiety and distress about the possibility of making a mistake or causing harm (Kaldal et al. 2018; McCarthy et al. 2018; Suikkala and Leino-Kilpi 2001). Furthermore, the presence of a preceptor, although appreciated by patients, may distract the student from focusing on the patient, especially if the student feels unsupported or has to prove the necessary competencies in nursing procedures according to the preceptor’s expectations (McCarthy et al. 2018; Suikkala et al. 2018).

Nurses, nursing students and patients do not necessarily view the nurse–patient relationship in the same way (Halldorsdottir 2008; Suikkala et al. 2008). We therefore need to study the congruence between student and patient views regarding their student–patient relationships. The purpose of this study was to analyse the congruence between matched student–patient pairs’ self-assessed views of their relationship during students’ clinical placement. The results of this study can be used for evaluating the processes and outcomes of clinical education.

Our basic assumption was that the higher the congruence between nursing students’ and patients’ views of student–patient relationships, the more mutual understanding the students and patients have of their assessments regarding their relationships. A common perception concerning the student–patient relationship is that at its best, knowledge and communication is shared by the patient and student through dialogue, benefiting both.

The following research questions were explored:

1. How congruent are the views of nursing students and patients regarding the student–patient relationship?
2. What factors shape the congruence between nursing students’ and patients’ views of their relationships?

## Methods

### Setting, sample and data collection

This study was conducted in Finland. The content, placements and time spent in clinical practice during nursing education (Bachelor’s degree, 3.5 years) are regulated by the European Union Directives (Directive 2005/36/EC, 2013/55/EU), the European Qualifications Framework (European Commission 2008) and national recommendations. Nursing training is carried out in 21 universities of applied sciences (UAS; Ministry of Education and Culture 2018). Clinical placements are organised in collaboration with various health care organisations (Maassen et al. 2011, p. 12); however, the UASs have legitimate autonomy to decide how clinical training is carried out in health care organisations such as hospital wards, outpatient clinics, and public health care services in the community, for instance (Lahtinen et al. 2014; Universities of Applied Sciences Act 932/2014).

The study used a cross-sectional survey design. The data consisted of a convenience sample of Finnish-speaking nursing students from six UASs representing the whole country geographically. A total of 1244 Finnish-speaking students in their first, second and graduating year were invited to undertake self-assessments of the student–patient relationship. The contact person in each UAS was informed about the subject of the study and asked to arrange distribution of the electronic questionnaire to students via e-mail during students’ penultimate week of clinical placement.

For collection of patient data, paper questionnaires, informed consent forms for patients, and written information about the research to students’ preceptors were provided to students with the assistance of UASs’ contact persons. In the clinical placement, students asked their preceptors to distribute the questionnaires to patients during the final two weeks of their clinical placement. Before handing out the questionnaires, preceptors asked patients meeting the sampling criteria to give their written consent to participate. Each student’s preceptor was requested to recruit one patient based on the following selection criteria: patients had to be aged 18 or over, Finnish-speaking, voluntarily participating in research, capable of answering the questionnaire, and involved in this particular student’s clinical education. A total of 288 patients met the selection criteria and were invited to participate.

In order to match students with the patients they were assigned to, each patient received a questionnaire labelled with the same unique ID code number that was given to the student who entered the electronic questionnaire. Completed questionnaires were received from 852 students and 272 patients. Of these, the final number of 187 students (response rate 21.9%) and 187 patients (response rate 64.9%) was matched for the sample of this study and included in the analysis.

### The student–patient relationship scale

The student–patient relationship scale (SPR scale, Suikkala 2007), developed based on a literature review (Suikkala and Leino-Kilpi 2001) and an interview study (Suikkala and Leino-Kilpi 2005), consists of 67 items structured to self-ratings divided into three types of relationship (items n = 33), contextual factors associated with the relationship (items n = 21), and the consequences of the relationship (n = 13). The three types of relationships are Mechanistic (MR), Authoritative (AR), and Facilitative (FA) relationships. Of these relationships, facilitative relationship can be seen as the priority which must be pursued between the student and the patient during clinical placements (Suikkala 2007; Suikkala et al. 2018). Furthermore, the contextual factors associated with the relationship are divided into Student’s personal and professional attributes (SA), Patient’s attributes as a patient (PA), and Atmosphere during collaboration (AC). Additionally, the consequences of the relationship are divided into Student’s personal and professional growth (SG), Student’s increased confidence and self-esteem (SC), and Patient’s improved health and commitment to self-care (PH). The questionnaires included parallel items for students and patients and were rated using a five-point Likert-scale (1 = strongly disagree, 5 = strongly agree).

The student questionnaire also included items on sociodemographic background (age, gender, education, and working experience before nursing education), current nursing education (current year of study, duration of clinical placement, and assessment of supervised clinical placement), student’s caring experiences (assignment to a specific patient, enough time for the patient, support received in relationship with patient, and experience of caring for ill family member), and perception of future nursing context of practice. The patient questionnaire included items on sociodemographic background (age, gender, education, and marital status), patient’s previous hospitalisation or other institutional care, reason for hospital or other institutional care admission, experienced state of health, size of patient room, and patient’s experience of caring (previous experience of student participation in care, named nursing student, student with enough time for the patient, experience of caring for ill family member).

For this study, internal consistencies (Cronbach’s alpha) of the scales indicated acceptable reliability (.70), with some exceptions presented in Table 1. The alpha values of the scales measuring mechanistic, authoritative and facilitative relationships ranged from good (.83) to questionable (.60), and the reliability coefficients of the factors associated with the relationship also ranged from good (.87) to questionable (.57) (Tavakol and Dennick 2011).

**Table 1:** Cronbach alpha reliability coefficients of the scales measuring mechanistic, authoritative and facilitative relationships and the factors associated with the relationships

Sum variables	Cronbach’s alpha		Number of items
	Students n = 187	Patients n = 187	
Mechanistic relationship	.59	.60	9
Authoritative relationship	.67	.80	11
Facilitative relationship	.79	.82	13
Student’s personal and professional attributes	.86	.77	8
Patient’s attributes as a patient	.57	.71	8
Atmosphere during activity	.80	.75	5
Student’s personal and professional growth	.80	.83	4
Student’s increased confidence and self-esteem	.75	.87	4
Patient’s improved health and commitment to self-care	.76	.84	5

The internal consistencies (Cronbach’s alpha .70 or above) of the scales indicated acceptable reliability, with some exceptions

### Data analysis

Data analysis was carried out using SPSS 25.0 (SPSS Inc., Chicago, USA). The data were analysed using descriptive statistics (frequencies, percentages, mean values, SDs, minimums, maximums). Congruent and non-congruent groups were compared for background variables using Chi square tests, Fisher’s exact tests and two-sample T-tests. Paired t-tests were performed to compare sum variable means between patients and students (Kim 2015). Wilcoxon signed ranks tests were used to compare patients and students for averages of individual items (Arora and Malhan 2010, pp. 465–469). Students and patients were matched in pairs and the Goodman’s and Kruskal’s Gamma coefficients (G) were used to evaluate congruence in individual items. The guideline used for interpretation of Gamma coefficient (G) was: G > .5 strong, .3 < G ≤ .5 moderate, .1 < G ≤ .3 weak (Adeyemi 2011). Binary logistic regression model was used to predict if students and patients had evaluated the student–patient relationship (0 = not equal, 1 = equal) as being exactly equal. Individual binary logistic models were used for background variables of patients and students (Peng et al. 2002).

### Ethics

The study protocol was approved by the Helsinki and Uusimaa University Hospital Ethical Committee (185/13/03/01/2014, 13.08.2014) and the authorities of all relevant health care organisations and UASs according to their standards. Both students and patients were informed about the study and received an information letter before volunteering and signing their consents. Anonymity, confidentiality, and the right to drop out at any stage of the study were guaranteed.

## Results

### Participant demographics

The mean age of the students was 29.7 years and the majority (87.3%) were female, representing Bachelor of Health Care degree (registered nurse) attained during statistical years 2016‒2018 when compared for age and gender (Vipunen - Education Statistics Finland 2020). About two-thirds (59.9%) had previous qualifications in social and health care and about half (54.6%) had previous nursing care experience. The number of first, second and third year was quite similar (range 23‒29). About three in four (78.1%) had a clinical placement lasting under six weeks and most assessed their clinical placement as inspiring (89.4%). Half of the students (54.1%) had an idea of the nursing context in which they would like to work in the future. Half of the students (51.6%) were assigned to a specific patient and most of the students (81.9%) had enough time for the patient. In most cases, teachers or supervising nurses (91.4%) had supported students in their relationships with patients. Half of the students (52.8%) had experience of caring for ill family members at home. The sociodemographic variables of students with congruent and non-congruent views with their patient are presented in more detail in Table 2. There were no significant differences between congruent and non-congruent views of their relationship with the patient with respect to the breakdown of sociodemographic variables (Table 2).

**Table 2:** Demographic information and *p* values for congruent and non-congruent views of all students and students in mechanistic, authoritative and facilitative relationships

	All students							Students in mechanistic relationships						
	Congruent			Non-congruent			*p* Value*	Congruent			Non-congruent			*p* Value*
Background variables	n	%	Mean (SD)	n	%	Mean (SD)		n	%	Mean (SD)	n	%	Mean (SD)	
*Age*	78		29.4 (9.6)	109		30.0 (9.3)	.694	147		30.0 (9.6)	40		29.0 (8.8)	.529
*Gender*							.135							.855
Male	7	9.0		18	16.5			20	13.6		5	12.5		
Female	71	91.0		91	83.5			127	86.4		35	87.5		
*Education*							.342							.669
Senior secondary/matriculation	34	43.6		40	36.7			57	38.8		17	42.5		
Social or health care	44	56.4		69	63.3			90	61.2		23	57.5		
*Working experience in nursing care*							.892							.948
Yes	35	44.9		50	45.9			67	45.6		18	45.0		
No	43	55.1		59	54.1			80	54.4		22	55.0		
*Experience of caring for ill family member*							.528							.711
Yes	43	55.1		55	50.5			76	51.7		22	55.0		
No	35	44.9		54	49.5			71	48.3		18	45.0		
*Current years of studies*							.174							.824
1st year	26	33.3		51	46.8			59	40.1		18	45.0		
2nd year	29	37.2		34	31.2			51	34.7		12	30.0		
3rd or 4th year	23	29.5		24	22.0			37	25.2		10	25.0		
*Duration of clinical placement*							.402							.122
2–5 weeks	59	75.6		88	80.7			112	76.2		35	87.5		
6–8 weeks	19	24.4		21	19.3			35	23.8		5	12.5		
*Assessment of supervised clinical placement*							.870							.873
Inspiring	70	89.7		97	89.0			131	89.1		36	90.0		
Frustrating	8	10.3		12	11.0			16	10.9		4	10.0		
*Assigned to a specific patient*							.194							.058
Yes	44	56.4		51	46.8			80	54.4		15	37.5		
No	34	43.6		58	53.2			67	45.6		25	62.5		
*Having enough time for the patient*							.944							.085
Yes	64	82.1		89	81.7			124	84.4		29	72.5		
No	14	17.9		20	18.3			23	15.6		11	27.5		
*Support received from*							.844							.704
Teacher/supervising nurse	72	91.1		100	91.7			136	91.9		36	90.0		
Other person within or outside the ward	7	8.9		9	8.3			12	8.1		4	10.0		
*Perception of future nursing context of practice*							.328							.393
Yes	45	57.7		55	50.5			81	55.1		19	47.5		
No	33	42.3		54	49.5			66	44.9		21	52.5		

	Students in authoritative relationships							Students in facilitative relationships						
	Congruent			Non-congruent			*p* Value*	Congruent			Non-congruent			*p* Value*
Background variables	n	%	Mean (SD)	n	%	Mean (SD)		n	%	MEAN (SD)	n = 82	%	mean (SD)	
*Age*	97		29.9 (9.6)	90		29.7 (9.3)	.885	105		29.1 (9.5)	82		30.6 (9.5)	.313
*Gender*							.397							.080
Male	11	11.3		14	15.6			10	9.5		15	18.3		
Female	86	88.7		76	84.4			95	90.5		67	81.7		
*Education*							.908							.101
Senior secondary/matriculation	38	39.2		36	40.0			47	44.8		27	32.9		
Social or health care	59	60.8		54	60.0			58	55.2		55	67.1		
*Working experience in nursing care*							.393							.270
Yes	47	48.5		38	42.2			44	41.9		41	50.0		
No	50	51.5		52	57.8			61	58.1		41	50.0		
*Experience of caring for ill family member*							.222							.560
Yes	55	56.7		43	47.8			57	54.3		41	50.0		
No	42	43.3		47	52.2			48	45.7		41	50.0		
*Current years of studies*							.197							.254
1st year	34	35.1		43	47.8			38	36.2		39	47.6		
2nd year	37	38.1		26	28.9			37	35.2		26	31.7		
3rd or 4th year	26	26.8		21	23.3			30	28.6		17	20.7		
*Duration of clinical placement*							.929							.896
2–5 weeks	76	78.4		71	78.9			83	79.0		64	78.0		
6–8 weeks	21	21.6		19	21.1			22	21.0		18	22.0		
*Assessment of supervised clinical placement*							.767							.288
Inspiring	86	88.7		81	90.0			96	91.4		71	86.6		
Frustrating	11	11.3		9	10.0			9	8.6		11	13.4		
*Assigned to a specific patient*							.614							.625
Yes	51	52.6		44	48.9			55	52.4		40	48.8		
No	46	47.4		46	51.1			50	47.6		42	51.2		
*Having enough time for the patient*							.809							.135
Yes	80	82.5		73	81.1			82	78.1		71	86.6		
No	17	17.5		17	18.9			23	21.9		11	13.4		
*Support received from*							.483							.297
Teacher/supervising nurse	91	92.9		81	90.0			95	89.0		77	93.9		
Other person within or outside the ward	7	7.1		9	10.0			11	10.4	10.4	5	6.1		
*Perception of future nursing context of practice*							.359							.965
Yes	55	56.7		45	50.0			56	53.3		44	53.7		
No	42	43.3		45	50.0			49	46.7		38	46.3		

*Congruent and non-congruent groups were compared for categorical background variables using Chi square tests and Fisher’s exact tests. Congruent and non-congruent groups were compared for numerical background variables using two-sample T-tests. The significance level for *p* values was set at .05

The mean age of the patients was 64 years. Over two-thirds of the patients were female (68.9%), two-fifths were (40.8‒46.7%) married/cohabiting (41.7%), and two-thirds (63.8%) had upper secondary or post-secondary non-tertiary education. Over two-thirds (66.8%) were elective patients, three-fourths (74.2%) had experienced three or more previous hospitalisations or other institutional care periods, and most (90.0%) assessed their state of health as at least moderate. About half (55.1–59.1%) shared a room with two or more patients. Over two-thirds of the patients (65.5%) had previous experience of student participation in care. Only about two-fifths of the patients (39.5%) reported being cared for by a named nursing student; however, most of the patients (93.4%) reported that the student had had enough time for them. Two-fifths of the patients (39.9%) had experience of caring for ill family members at home. The sociodemographic variables of the patients with congruent and non-congruent views with their student are presented in more detail in Table 3. For the patients, there was only one significant difference with respect to the breakdown of sociodemographic variables: the patients with congruent views with students in facilitative relationship were more often male (*p* = .039) (Table 3).

**Table 3:** Demographic information and *p* values for congruent and non-congruent views of all patients and patients in mechanistic, authoritative and facilitative relationships

Background variables	All patients							Patients in mechanistic relationships						
	Congruent			Non-congruent			*p* Value*	Congruent			Non-congruent			*p* Value*
	n	%	Mean (SD)	n	%	Mean (SD)		n	%	Mean (SD)	n	%	Mean (SD)	
*Age*	78		61.7 (18.6)	106		66.3 (16.7)	.083	147		63.4 (18.1)	40		68.2 (16.1)	.130
*Gender*							.074							.238
Male	28	37.3		27	25			46	32.2		9	22.5		
Female	24	62.7		81	75			97	67.8		31	77.5		
*Education*							.368							.910
Basic education	24	31.2		45	41.3			53	36.3		16	40.0		
Secondary level qualifications	42	54.5		50	45.9			73	50.0		19	47.5		
Higher education	11	14.3		14	12.8			20	13.7		5	12.5		
*Marital status*							.385							.848
Married/cohabiting	35	44.9		42	38.5			60	40.8		17	42.5		
Not married/not cohabited	43	55.1		67	61.5			87	59.2		23	57.5		
*Previous hospitalisations or other institutional care*							.648							.987
Two or less	21	27.3		21	24.3			37	25.5		10	25.5		
Three or more	56	72.7		81	75.7			108	74.5		29	74.4		
*Reason for hospital or other institutional care admission*							.685							.476
Elective patient	51	65.4		73	68.2			96	65.8		28	71.8		
Emergency patient	27	34.6		34	31.8			50	34.2		11	28.2		
*Experienced state of health*							.796							.983
Very good	3	3.8		5	4.7			6	4.1		2	5.0		
Good	15	19.0		15	14.0			24	16.4		6	15.0		
Moderate	44	55.7		66	61.7			86	58.9		24	60.0		
Poor	31	16.7		18	16.8			25	17.1		6	15.0		
Very poor	7	3.8		3	2.8			5	3.4		2	5.0		
*Caring environment*							.727							.888
One-patient room	17	21.5		27	25.2			35	23.8		9	22.0		
More than one patient in room	44	55.7		60	56.1			81	55.1		23	58.0		
Home	18	22.8		20	18.7			31	21.1		8	20.0		
*Previous experience of student participation in care*							.650							.735
Yes	53	67.1		69	63.9			95	64.6		27	67.5		
No	26	32.9		39	36.1			52	35.4		13	32.5		
*Named nursing student*							.286							.005
Yes	33	43.4		37	35.6			62	44.3		8	20.0		
No	43	56.6		67	64.4			78	55.7		32	80.0		
*Student has enough time for the patient*							.144							.034
Yes	75	96.2		96	90.6			138	95.2		33	84.6		
No	3	3.8		10	9.4			7	4.8		6	15.4		
*Experience of caring for ill family member*							.748							.751
Yes	32	41.0		41	38.7			58	40.3		15	37.5		
No	46	59.0		65	61.3			86	59.7		25	62.5		

Background variables	Patients in authoritative relationships							Patients in facilitative relationships						
	Congruent			Non-congruent			*p* Value*	Congruent			Non-congruent			*p* Value*
	n	%	Mean (SD)	n	%	Mean (SD)		n	%	Mean (SD)	n	%	Mean (SD)	
*Age*	96		63.3 (18.3)	88		65.6 (17.1)	.379	104		63.6 (18.2)	80		65.4 (17.1)	.508
*Gender*							.573							**.039**
Male	30	31.9		25	28.1			37	36.3		18	22.2		
Female	64	68.1		64	71.9			65	63.7		63	77.8		
*Education*							.346							.906
Basic education	31	32.3		38	42.2			38	36.6		31	37.8		
Secondary level qualifications	52	54.2		40	44.4			51	49.0		41	50.0		
Higher education	13	13.5		12	13.3			15	14.4		10	12.2		
*Marital status*							.986							.084
Married/cohabiting	40	41.2		37	41.1			49	46.7		28	34.1		
Not married/not cohabited	57	58.8		53	58.9			56	53.3		54	65.9		
*Previous hospitalisations or other institutional care*							.804							.625
Two or less	25	26.3		22	24.7			28	26.9		19	23.8		
Three or more	70	73.3		67	75.3			76	73.1		61	76.3		
*Reason for hospital or other institutional care admission*							.345							.470
Elective patient	62	63.9		62	70.5			72	69.2		52	64.2		
Emergency patient	35	36.1		26	29.5			32	30.8		29	35.8		
*Experienced state of health*							.138							.835
Very good	3	3.1		5	5.7			6	5.7		2	2.5		
Good	20	20.4		10	11.4			18	17.0		12	15.0		
Moderate	54	55.1		56	63.3			60	56.6		50	62.5		
Poor	15	15.3		16	18.2			18	17.0		13	16.3		
Very poor	6	6.1		1	1.1			4	3.8		3	3.8		
*Caring environment*							.998							.346
One-patient room	23	23.5		21	23.9			21	20.0		23	28.4		
More than one patient in room	55	56.1		49	55.7			60	57.1		44	54.3		
Home	20	20.4		18	20.5			24	22.9		14	17.3		
*Previous experience of student participation in care*							.346							.793
Yes	67	68.4		55	61.6			70	66.0		52	64.2		
No	31	31.6		34	38.2			36	34.0		29	35.8		
*Named nursing student*							.772							.770
Yes	36	37.9		34	40.0			41	39.8		29	27.3		
No	59	62.1		51	60.0			62	60.2		48	62.3		
*Student has enough time for the patient*							.652							.410
Yes	90	93.8		81	92.0			99	94.3		72	91.1		
No	6	6.3		7	8.0			6	5.7		7	8.9		
*Experience of caring for ill family member*							.884							.476
Yes	38	39.2		35	40.2			44	44.9		29	36.7		
No	59	60.8		52	59.8			61	58.1		50	63.3		

*Congruent and non-congruent groups were compared for categorical background variables using Chi square tests and Fisher’s exact tests. Congruent and non-congruent groups were compared for numerical background variables using two-sample T-tests. The significance level for *p* values was set at .05. Values of significance < .05 have been bolded

### The congruence of nursing students’ and patients’ views of their relationships

In both students’ and patients’ assessments, authoritative and facilitative relationships were prominent. Students viewed the relationship more often as facilitative and authoritative than mechanistic. In the patients’ assessments, authoritative relationship occurred most frequently, followed by facilitative and mechanistic relationship. Furthermore, students’ sum variable mean value for facilitative relationship was significantly higher (FA, *p* < .001) than the corresponding mean value of patients. For mechanistic relationship, students’ sum variable mean value was significantly lower (MR, *p* < .001) than the corresponding mean value of patients. At item level (significance level *p* < .05), students’ and patients’ views of their relationship are presented in Table 4.

**Table 4:** The congruence between students’ and patients’ views of mechanistic, authoritative and facilitative relationships (5-point Likert-type scale, 1 = totally disagree, 5 = totally agree)

Sum variables and questions concerning	Patients			Students			Z^1^	*p* Value^a^	Valid cases	Gamma value^b^	Asymptotic standard error^2^	Appropriate T^3^	*p* Value^b^
	N	Mean	SD	n	Mean	SD							
*Mechanistic relationship (MR)*	186	3.41	.61	186	3.11	.51		**< .001**					
Focused on student learning	183	4.25	.93	187	3.95	.95	− 3.043	**.002**	183	.066	.099	.671	.502
Externally directed by supervising nurse’s actions	183	4.40	1.03	187	4.47	.87	− .468	.640	183	.315	.113	2.446	**.014**
Student and patient do not know each other	184	2.20	1.28	187	2.06	1.08	− 1.306	.192	184	.082	.087	.940	.347
Student’s attention focused on technical performance	184	3.00	1.40	187	2.19	.98	− 6.057	**< .001**	184	.106	.084	1.266	.205
Negligible discussion between student and patient	184	2.30	1.26	187	1.90	.98	− 3.740	**< .001**	184	.184	.085	2.115	**.034**
Student observes nurse’s actions	186	3.96	1.08	187	3.87	1.27	− .676	.499	186	.133	.092	1.430	.153
Student imitates nurse’s actions	185	3.92	1.54	187	4.08	.88	− 1.749	.080	185	.284	.097	2.812	**.005**
Patient is passive object of nursing actions	184	3.64	1.33	187	3.21	1.23	− 3.078	**.002**	184	.124	.82	1.510	.131
Patient observes student’s actions	184	2.94	1.43	187	2.27	1.21	− 4.638	**< .001**	184	.077	.076	1.018	.309
*Authoritative relationship (AR)*	187	3.92	.65	187	4.00	.44		.155					
Focused on assumptions of what is best for the patient	182	4.23	.97	187	4.07	.97	− 1.541	.123	182	.051	.106	.478	.633
Patient care decisions taken by student	183	3.45	1.19	187	3.16	1.13	− 2.275	**.023**	183	.221	.073	2.996	**.003**
Student knows the patient as a patient with a certain disease	183	3.73	1.25	187	2.94	1.13	− 6.045	**< .001**	183	.095	.086	1.105	.269
Conversation on care-related issues	184	3.98	1.20	187	4.17	.87	− 1.964	**.050**	184	.195	.101	1.897	.058
Conversation on everyday issues character	183	3.79	1.24	187	4.46	.85	− 6.342	**< .001**	183	.320	.092	3.214	**.001**
Student helps patient to the best of her ability	185	4.79	.48	187	4.86	.38	− 1.463	.143	185	.197	.233	.747	.455
Student provides daily care	186	4.35	.99	187	4.51	.83	− 2.101	**.036**	186	.242	.119	1.862	.063
Student clarifies what is best for the patient	186	4.05	1.06	187	4.10	.82	− .306	.760	186	.084	.099	.848	.396
Student activates patient in self-care	184	3.68	1.27	187	4.10	.95	− 3.569	**< .001**	184	.137	.088	1.553	.120
Patient asks student for advice	183	3.44	1.32	187	3.77	1.06	− 2.795	**.005**	183	.188	.084	2.208	**.027**
Patient agrees to student’s suggestions	184	3.55	1.18	187	3.83	.80	− 2.877	**.004**	184	.285	.083	3.318	**.001**
*Facilitative relationship (FR)*	187	3.72	.69	187	4.04	.51		**< .001**					
Focused on the common good of both student and patient	186	4.65	.62	187	4.56	.64	− 1.254	.210	186	.166	.147	.774	.439
Directed by patient’s wishes	184	4.35	.82	187	4.40	.59	− .692	.489	184	− .172	.119	− 1.427	.153
Student and patient know each other personally	183	3.85	1.12	187	3.85	1.01	− .261	.794	183	.225	.085	2.603	**.009**
Conversation on confidential matters	184	3.64	1.31	187	3.79	1.10	− 1.294	.196	184	.314	.078	.3.969	**< .001**
Conversation on patient’s emotions	182	3.34	1.30	187	3.95	1.04	− 5.513	**< .001**	182	.265	.082	3.178	**.001**
Student listens to the patient	184	4.13	1.12	187	4.51	.71	− 4.050	**< .001**	184	.293	.102	2.664	**.008**
Student acts as an advocate for patient	186	3.65	1.28	187	3.99	1.23	− 3.039	**.002**	186	.200	.090	2.174	**.030**
Student encourages patient	182	4.23	1.01	187	4.47	.71	− 2.829	**.005**	182	.063	.117	.534	.594
Patient is expert of own situation	184	3.61	1.21	187	4.01	.86	− 3.604	**< .001**	184	.147	.089	1.648	.099
Patient expresses opinions to student in care-related matters	180	3.42	1.31	187	4.18	.91	− 6.242	**< .001**	180	.199	.084	2.345	**.019**
Patient provides information to student in matters related to disease	182	3.35	1.28	187	4.01	.98	− 5.545	**< .001**	182	.187	.088	2.111	**.035**
Patient gives advice to student	182	2.52	1.38	187	2.99	1.23	− 3.509	**< .001**	182	.245	.076	3.183	**.001**
Patient gives feedback to student	183	3.40	1.48	187	3.86	1.13	− 3.703	**< .001**	183	.255	.082	3.050	**.002**

^a^Paired t-tests were performed to compare sum variable means between patients and students. Wilcoxon signed ranks tests were used to compare patients and students for averages of individual items. Significantly higher mean values than the corresponding ones have been underlined

^b^Goodman’s and Kruskal’s Gamma coefficients (G) were used to evaluate congruence in individual items of matched pairs of students and patients. Interpretation of Gamma coefficient (G) was: G > .5 strong, .3 < G ≤ .5 moderate, .1 < G ≤ .3 weak

^a.b^The significance level for *p* values was set at .05. Values of significance < .05 have been bolded

^1^Asymptotic value from standardized normal distribution

^2^Not assuming the null hypothesis

^3^Using the symptotic standard error assuming the null hypothesis

As a whole, 78 out of 187 student–patient pairs viewed their relationship congruently. In a more detailed analysis, 148 students viewed the mechanistic relationship, 105 the facilitative relationship, and 98 the authoritative relationship congruently with their patients (Tables 2, 3). Furthermore, students and patients had moderately (G = .315 − .320) to weakly (G = .106 − .293) congruent views on facilitative relationship (FA, 9 out of 13 items), authoritative relationship (AR, 4 out of 11 items, and mechanistic relationship (2 out of 9 items). Moderate to weak positive congruence between students’ and patients’ views at significance level *p* < .05 is presented in more detail in Table 4.

### Factors associated with the congruence between nursing students’ and patients’ views of their relationship

Students’ and patients’ assessments of the contextual factors and consequences of the relationship showed that, in general, both had very positive perceptions (mean > 4.0) of student’s personal and professional attributes (SA), the atmosphere during collaboration (AC), student’s personal and professional growth (SG), and student’s increased confidence and self-esteem (SC). Patients assessed the contextual factors and consequences of the relationship significantly higher than students in the sum variables of patient’s attributes as a patient (PA, *p* = .013) and patient’s improved health and commitment to self-care (PH, *p* < .001) whereas the opposite was true for student’s personal and professional growth (PG, *p* < .001) and student’s increased confidence and self-esteem (SC, *p* < .001). Students’ and patients’ views at item level are presented in more detail in Table 5.

**Table 5:** The congruence of students and patients views of contextual factors and consequences of the relationships (5-point Likert-type scale, 1 = totally disagree, 5 = totally agree)

Sum variables and questions concerning	Patients			Students			Z^1^	*p* Value^a^	Valid cases	Gamma value^b^	Asymptotic standard error^2^	Appropriate T^3^	*p* Value^b^
	n	Mean	SD	n	Mean	SD							
*Student’s personal and professional attributes*	186	4.57	.51	186	4.60	.34		.421					
Sense of humour	186	4.51	.75	187	4.53	.58	− .452	.652	186	− 037	.135	− .276	.782
Empathy	186	4.65	.68	187	4.68	.49	− .356	.722	186	.347	.140	2.139	**.032**
Ability to deal with patient feedback	183	4.29	.88	187	4.64	.56	− 4.407	**< .001**	183	.151	.127	1.152	.249
Ability to remain calm	184	4.80	.50	187	4.79	.43	− .174	.862	184	.113	.224	.475	.634
Recognising the patients as an equal partner	186	4.77	.57	187	4.86	.36	− 1.842	.066	186	.229	.229	.859	.390
Ability to answer patient’s questions	184	4.32	.86	187	3.98	.74	− 3.994	**< .001**	184	.065	.108	.599	.549
Ability to perform care-related activities with care	185	4.68	.65	187	4.74	.49	− .844	.399	185	− .016	.190	− .082	.934
Ability to discuss issues in a natural, unforced manner	182	4.49	.83	187	4.56	.63	− 1.019	.308	182	.196	.129	1.429	.153
*Patient’s attributes as a patient*	187	3.98	.64	187	3.84	.50		**.013**					
Willingness to talk about one’s own situation	184	4.24	.96	187	4.38	.74	− 1.423	.155	184	.211	.103	1.987	**.047**
Sense of humour	185	4.29	.88	187	4.49	.73	− 2.965	**.003**	185	.373	.102	3.350	**.001**
Physical condition	182	3.51	1.22	187	3.56	1.09	− .563	.574	182	.031	.083	.367	.714
Mood	183	3.96	1.01	187	3.80	1.04	− 1.662	.097	183	.303	.088	3.293	**.001**
Need for help with daily activities	185	2.49	1.43	187	2.59	1.35	− .956	.339	185	.456	.064	6.834	**< .001**
Attitude to student	185	4.44	.91	187	4.30	.80	− 2.059	**.039**	185	.157	.114	1.335	.182
Attitude to student learning	183	3.37	1.40	187	2.60	1.21	− 5.754	**< .001**	183	.254	.079	3.153	**.002**
Commitment to self-care	184	4.41	.88	187	4.20	.82	− 2.563	**.010**	184	.116	.113	1.019	.308
*Atmosphere during activity*	181	4.45	.60	181	4.37	.61		.162					
Staff nurses’ performance as role models	181	**4.73**	.54	187	4.35	.76	− 5.365	**< .001**	181	.339	.128	2.391	**.017**
Staff nurses’ attitudes towards students	180	**4.67**	.58	187	4.45	.75	− 2.624	**.009**	180	.024	.141	.167	.868
Staff nurses’ supportive supervision	180	4.49	.79	187	4.39	.88	− .889	.374	180	.267	.099	2.571	**.010**
Staff nurses’ encouraging feedback	180	4.24	.91	187	4.29	.88	− .772	.440	180	.231	.115	1.875	.061
Privacy for student–patient interaction	180	4.10	1.23	187	4.27	.99	− 1.798	.072	180	.255	.100	2.144	**.032**
*Student’s personal and professional growth*	177	4.17	.71	177	4.51	.51		**< .001**					
Growth as human being	176	3.95	.90	187	4.24	.80	− 2.887	**.004**	176	.092	.093	.985	.325
Growth as nurse	177	4.26	.83	187	4.63	.58	− 4.563	**< .001**	177	.206	.120	1.654	.098
Learned to respect the patient as unique personality	176	4.41	.80	187	4.65	.59	− 2.906	**.004**	176	.060	.134	.441	.659
Expanded knowledge base in patient illness and care	177	4.04	.96	187	4.56	.59	− 5.868	**< .001**	177	.128	.144	1.113	.266
*Student’s increased confidence and self-esteem*	177	4.34	.69	177	4.59	.50		**< .001**					
Successful advocacy of what is best for the patient	175	4.29	.85	187	4.70	.57	− 5.257	**< .001**	175	.366	.115	2.801	**.005**
Successful involvement in caring relationship with patient	176	4.31	.79	187	4.60	.68	− 3.656	**< .001**	176	.307	.110	2.590	**.010**
Confidence to face new situations	177	4.28	.83	187	4.65	.59	− 4.687	**< .001**	177	.182	.120	1.466	.143
Motivation to pursue nursing as a career	175	4.49	.77	187	4.45	.77	− .913	.361	175	.264	.113	2.171	**.030**
*Patient’s improved health and commitment to self-care*	181	4.21	.76	181	3.86	.63		**< .001**					
Increased sense of health and well-being	181	4.45	.82	187	3.77	1.00	− 6.066	**< .001**	181	.063	.104	.607	.544
Alleviation of tension	179	4.26	.86	187	3.70	.94	− 5.413	**< .001**	179	.094	.102	.927	.354
Increased knowledge about one’s own care	178	3.77	1.16	187	3.65	.91	− 1.033	.302	178	.143	.088	1.619	.106
Receiving help whenever necessary	181	4.48	.94	187	4.43	.70	− 1.144	.253	181	.245	.116	2.000	**.046**
Increased compliance	179	4.04	1.10	187	3.71	.88	− 3.665	**< .001**	179	.305	.087	3.396	**.001**

^a^Paired t-tests were performed to compare sum variable means between patients and students. Wilcoxon signed ranks tests were used to compare patients and students for averages of individual items. Significantly higher mean values than the corresponding ones have been underlined

^b^Goodman’s and Kruskal’s Gamma coefficients (G) were used to evaluate congruence in individual items of matched pairs of students and patients. Interpretation of Gamma coefficient (G) was: G > .5 strong, .3 < G ≤ .5 moderate, .1 < G ≤ .3 weak

^a.b^The significance level for *p* values was set at .05. Values of significance < .05 have been bolded

^1^Asymptotic value from standardized normal distribution

^2^Not assuming the null hypothesis

^3^Using the symptotic standard error assuming the null hypothesis

In contextual factors and consequences of the relationship, moderately (G = .303 − .456) to weakly (G = .113 − .267) congruent views were found on patient’s attributes as a patient (PA, 5 out of 9 items), atmosphere during activity (AC, 3 out of 5 items), student’s increased confidence and self-esteem (SC, 2 out of 4 items), and patient’s improved health and commitment to self-care (PH, 2 out of 5 items). Among these, moderately congruent assessments between students and patients were found in items describing student’s empathy (SA), patient’s sense of humour, mood and need for help with daily activities (PA), staff nurses’ performance as role models (AC), student’s successful advocacy of what is best for the patient, and student’s successful involvement in caring relationship with patient (SC). Weak positive congruence at significance level *p* < .05 is presented in more detail in Table 5.

Patients with congruent views in a mechanistic relationship assessed student’s personal and professional attributes (*p* = .015), student’s personal and professional growth (*p* = .001), student’s increased confidence and self-esteem (*p* = .002), patient’s attributes as a patient (*p* = .027), and patient’s improved health and commitment to self-care (*p* < .001) significantly higher than those with non-congruent views. The opposite was true for their views with students in an authoritative relationship as concerned atmosphere during collaboration (*p* = .035). Congruent views between students and patients in a facilitative relationship were seen less often among students who viewed their own personal and professional attributes lower than those with non-congruent views (*p* = .001).

Only two sociodemographic variables predicted congruence of students’ and patients’ views. In general, the older the patients, the more their views differed from those of the students (OR = .878, *p* = .026). Furthermore, elective patients (OR = .24, *p* = .038) and older patients (OR = .949, *p* = .010) were less likely to view the relationship as facilitative (OR = .949, *p* = .010).

## Discussion

In nursing education, there is an assumption that student–patient relationships benefit both learning and patient care, and that student and patient views on their relationships can provide valuable insights on the professional performance of students. By analysing the congruence between the views of matched nursing student and patient pairs of their relationship during the students’ clinical placement we found that student and patient views were moderately or weakly congruent in terms of facilitative relationship. Patients, however, tended to see the relationship significantly more often as mechanistic. Patient’s age and reason for care were the only background variables that predicted the congruence between students’ and patients’ views of their relationship.

Based on these results, it seems that nursing students realise the value of patients in their learning. This confirms the arguments that the value of student–patient relationships is better recognised in undergraduate education, and patient participation in competence-based clinical education is gradually expanding (Rowland et al. 2018; Scammell et al. 2016; Suikkala et al. 2018). However, the congruence was at best only moderate, which suggests that more work is needed on patient participation in clinical education. Patient views about the facilitative relationship might be due to unfamiliarity or inexperience concerning their active role in students’ learning process while hospitalised, e.g. by providing information to students on matters related to disease and giving advice to students. It is the shared responsibility of both educational institutions and health care organisations to provide clinical placements where both educational structures and the attitudinal atmosphere nurture reciprocal collaborative student–patient relationships as an integral part of the development of professional competence.

Students rated the facilitative and authoritative relationships highest (mean > 4.0 on the 5-point Likert scale). Although authoritative and mechanistic relationships offer opportunities to gain experience and confidence in performance, students will miss the holistic perspective of the patients and their situation if they do not reach and sustain facilitative levels of relationships with their patients. Although there were no statistically significant differences in the background variables on congruent and non-congruent views of students, final year students showed the lowest levels of mechanistic and authoritative relationships. This might indicate that students who are about to step into the world of work are better attuned to working in collaboration with patients. To some extent, this finding contradicts previous ones (Kaldal et al. 2018; McCarthy et al. 2018; Suikkala and Leino-Kilpi 2001) arguing that graduating students face inconvenience in establishing relationships with patients because of their feelings of incompetence. We think that we should support students, even near graduation, in establishing relationships with patients to help them gain confidence in patient care (Manninen 2014; Suikkala et al. 2008). Moreover, it is quite natural that students who have only recently started their education tend to focus on the nursing procedures. However, a mutual confidence-based relationship with good communication with patients is still considered as the essence of nursing practice among students (Bagdonaite-Stelmokiene et al. 2016).

Background factors explained some of the differences concerning the views of the relationships. Although a facilitative relationship characterised by mutuality can be a learning experience for students as well as for patients with various health conditions (Bleakley and Bligh 2008; Rowland et al. 2018; Suikkala and Leino-Kilpi 2005), not all seemed to find it the customary way to interact. Our results showed that the older the patients, the more their views differed from students’ views of their relationship. The reason for this can be rather conventional, as older patients are, arguably, more used to the paternalistic nature of care. The starting point in this kind of care relationship is not equal partnership, but rather, the dominance of the health care professionals (Angel and Frederiksen 2015; Grilo et al. 2014). With an aging population, it is advisable to consider how older people could be encouraged and supported to participate in student learning. Another group that held different views about student relationships were elective patients, perhaps because these patients expected their hospital stay to follow a certain care pathway with specified events which they are informed of in advance. Moreover, the current standards favouring short hospital stays following, for instance, a surgical procedure may also have influenced the development of reciprocal relationships with students as any nurse–patient-relationship requires some time to reach its fulfilment (Strandås and Bondas 2018). Any patient, regardless of their situation, who is able to participate in reciprocal dialogue with students influences and shapes the qualities of future professionals (Angel and Frederiksen 2015; Rowland et al. 2018).

Clinical learning environments that support facilitative relationships through dialogue between students and patients are of great importance and in line with a person-centred, evidence-based approach (Håkansson-Eklund et al. 2019; Jylhä et al. 2017; WHO 2015). The results of this study confirmed the significance of a positive atmosphere, and particularly, staff nurses’ performance as role models, with their supervision focused on supporting students in forming a relationship with patients and thus involving patients as part of the educational team (Ford et al. 2016; Suikkala et al. 2018; Manninen 2014). In previous studies, student–preceptor relationships have been identified as being of key importance in supporting and facilitating students to engage in relationships with patients as part of their process of becoming professional caregivers (Bombeke et al. 2010; Ford et al. 2016; Kaldal et al. 2018). Even preceptors feel that patients have valuable perspectives that enrich nursing students’ clinical learning and assessment; however, at the same time, they may worry about the ethical and practical challenges of patient involvement, especially if patient views differ from those of preceptors (Towle et al. 2010). In clinical placements, the relationship between students and their preceptor can often be seen as predominant, overshadowing patients as part of quality clinical placement experiences. Thus, it is also essential that there is a common understanding of the centrality of the student–patient relationships within the context of clinical education (Ford et al. 2016). In facilitative relationships, patients are seen as part of the educational team in which students and patients are engaged in reciprocally favourable dialogue supported by preceptors (Suikkala and Leino-Kilpi 2005; Manninen 2014).

The results of this study demonstrated patient contributions towards students’ professional development, such as personal and professional growth and improved confidence and self-esteem (Fröberg et al. 2018; McMahon-Parkes et al. 2016). This positive view of the consequences of the relationship for students was held more often by students than patients. Instead, improved state of health and commitment to self-care were more often seen as positive consequences by patients than by students. These findings show that the contribution of the student–patient relationship can be quite difficult to assess from one another’s perspective, but patient involvement in clinical education benefits both students and patients (Suikkala et al. 2018). Patients can benefit by gaining a greater understanding of their own situation and through this, by playing a more active role in their own care (Suikkala et al. 2018; Strömwall et al. 2018).

### Limitations

This study involves some limitations related to the samples and the instrument used. The sampling bias was reduced by choosing the UASs and clinical placements of students from different parts of Finland and by recruiting as many students and patients as possible who belonged to the study population. However, out of the total of 1244 recruited students, only 187 students formed matched pairs with their patients recruited from amongst those receiving care from these students. Based on the small sample size and potential selection bias caused by having only certain kinds of patients (mostly those in good health) participate in the study, questions about representativity can be raised as the results may to some extent be specific to the sample. For instance, the length of questionnaires and the time of completing a survey might have affected response rates for some patients (Fan and Yan 2010).

The SPR scales (Suikkala 2007) used in this study were theoretically derived from a literature review (Suikkala and Leino-Kilpi 2001) and an interview study (Suikkala and Leino-Kilpi 2005). Both of these focused on proving insight into the characteristics of the nursing student–patient relationship, factors determining the relationship, as well as the benefits of the relationship for both students and patients. The SPR scales have been tested and implemented for nursing students and patients in the internal medicine context in hospitals (Suikkala et al. 2007, 2008, 2009). In this study, the SPR scales (Suikkala 2007) were used for the first time in any clinical learning context students have during their education. Both students and patients were asked to answer honestly, emphasising the confidentiality of the answers. It is, however, noteworthy that self-administered SRP scales assess the students’ and patients’ perceptions, not how they actually behave or interact in their relationship. Thus, the assessments might be based on the ideal that students have been taught about their relationships with patients, and on the part of the patients, authentic patient experiences may have been influenced by socially desirable answers. Furthermore, the Gamma coefficients (G) measure the degree of agreement between students’ and patients’ views (Adeyemi 2011). Weakness of the Gamma coefficient (G) thus means that it cannot exactly represent the occurrence of congruence.

This study did not verify the benefits of the facilitative student–patient relationship for students’ learning, their academic performance, competence development, motivation to pursue a career in nursing or empowering the patient, or how their relationships empowered the patient or contributed to enhanced quality of care. However, considering moments of knowledge and communication shared by the patient and student through dialogue enabled mutual learning; even a patient without the expertise of experience was thus able to participate in a relationship that had beneficial consequences for both student learning and the well-being of the patient.

### Recommendations for practice

Moving the educational focus from the relationship between student and preceptor to the relationship between student and their patients has some potential implications for clinical placements and the supervision of students. Both educators in the university environment and preceptors in the clinical environment have the possibility to promote the success of students to learn with and from patients. Educators in universities should be familiar with clinical placements and provide their pedagogical expertise to students and clinicians in order to support students’ orientation towards facilitative relationships with patients. Educators can encourage and guide students to focus on genuine and empathic communication and to solicit and listen to patients’ personal viewpoint in encounters with patients. Preceptors in the clinical environment can foster the student–patient relationship by ensuring that the student has a reasonable amount and quality of time to get to know the patient as an individual person along with the nursing activities. Furthermore, as positive role-models and facilitators, preceptors and all nursing staff have an important role in assisting students to understand the core substance of individual patient encounters that benefit both students and patients.

## Conclusions

This study focused on the rarely studied area of the student–patient relationship in clinical placements in a preregistration nursing programme by combining the views of students and patients. The results show that students’ views on facilitative relationship with patients are quite in line with those of their patients. Hence, facilitative student–patient relationships highlighting patients as a core of student learning can be important contributors to student clinical learning. This can be achieved by ensuring meaningful student–patient dialogue, and thus integrating patients’ experiential knowledge and students’ academic learning through the support provided by health professionals and educators. While aiming to strengthen student–patient relationships in the future it is justified to focus on mutually beneficial relationships between students and patients to provide information for adjusting clinical supervision practices. The relevance of mutual learning of students and patients should also be considered.
